# Characterization of clock proteins in the substantia nigra and subthalamic nucleus of the *Sapajus apella* primate

**DOI:** 10.3389/fnana.2024.1480971

**Published:** 2024-11-13

**Authors:** Leila Maria Guissoni Campos, Gyovanna Sorrentino dos Santos Campanari, Jeferson Santiago, Eduardo Vinicius Barboza Santos, Alana Cristy Ghiraldelli Santos, Mayara Longui Cabrini, Mauro Audi, Isabela Bazzo Costa, Viviane Canhizares Evangelista de Araujo, Stephannie Monaco Bodra, Maressa Monteiro Pereira Gualassi, Lívia Clemente Motta-Teixeira, Luciana Pinato

**Affiliations:** ^1^Postgraduate Program in Structural and Functional Interactions in Rehabilitation – UNIMAR, School of Medicine, Universidade de Marília (UNIMAR), Marilia, Brazil; ^2^Santa Casa de São Paulo School of Medical Sciences, São Paulo, Brazil; ^3^Department of Speech, Language and Hearing Sciences, São Paulo State University (UNESP), Marilia, Brazil

**Keywords:** substantia nigra, subthalamic nucleus, clock genes, dopamine, circadian rhythms, primate

## Abstract

Clock genes, which are essential for suprachiasmatic nucleus (SCN) function, also play critical roles in other brain regions, and their expression have been the subject of various studies. An increasingly deeper understanding of the expression of these genes in different species contributes to our knowledge of their functions and the factors influencing their expression. Considering that most studies have been conducted in nocturnal rodents, in this study we investigated the presence of Per1, Per2 and Cry1 in neurons of the substantia nigra (SN) and subthalamic nucleus (STN) in a diurnal primate. The immunoreactivity of Per1, Per2, and Cry1 was analyzed using immunohistochemistry, revealing significant Per1-IR, Per2-IR, and Cry1-IR in the SN. While Per1-IR and Per2-IR were also observed in the STN, no Cry1-IR staining was detected in the STN. These results confirm the presence of proteins that regulate circadian rhythms in areas associated with motor behavior.

## 1 Introduction

Numerous cellular functions and behaviors exhibit temporal variations that are critical for adaptation to daily and seasonal environmental changes. These variations are governed by circadian rhythms that respond to environmental changes through the expression of clock genes (Hauber and Bareiss, 2001; Smarr et al., 2014; Hastings et al., 2019).

The temporal variations are modulated by a circadian timing system composed of oscillators, modulating structures and synchronizing pathways. In mammals, the main neural components of this system are the suprachiasmatic nucleus (SCN) of the hypothalamus, the dominant central circadian pacemaker, the retinohypothalamic, geniculohypothalamic (GHT) and raphe-SCN pathways. The GHT originates from the intergeniculate wing, which appears to be part of the pregeniculate nucleus in primates (Reuss, 1996; Cavalcante et al., 2002; Morin and Allen, 2006).

Cells of the SCN express autonomous rhythmic gene activity driven by transcriptional regulation of clock genes, with CRY/PER heterodimers acting as transcriptional repressors of their own genes (Reppert and Weaver, 2001). Thus, the SCN operates through a self-regulatory feedback mechanism that drives the rhythmic expression of these genes, including Bmal1, Per1, Per2, Per3, Cry1, Cry2, Clock, Arntl, and Nr1d1 (Lowrey and Takahashi, 2011).

The core circadian feedback loop formed by the interactions between these genes and proteins is as follows: CLOCK and BMAL1 bind to the promoter regions of Per and Cry, initiating the transcription of these genes; the protein products, PER and CRY, form a complex that enters the nucleus and represses the transcriptional activity of CLOCK and BMAL1, subsequently stopping the transcription of Per and Cry (Lowrey and Takahashi, 2011; Mohawk et al., 2012). This loop repeats every 24 h.

While it is clear that the expression of clock genes determines various biological rhythms, it is also known that hormones and neurotransmitters can modulate these rhythms, likely through the regulation of these genes. Dopamine (DA), a neurotransmitter known for its roles in locomotion, reward, and learning, is emerging as an important neuromodulator of both central and peripheral circadian rhythms, potentially influencing the expression of clock proteins (Webb et al., 2009; Hood et al., 2010; Imbesi et al., 2009).

The relationship between dopaminergic neurons and clock genes appears to be bidirectional. DA is relevant to the SCN because the main SCN clock communicates timing information with other brain clocks to regulate DA activity, and DA also appears to have feedback effects on the SCN (Mendoza and Challet, 2014). A role for dopaminergic modulation of SCN-related circadian rhythmicity is suggested by the presence of D1 and D5 receptors in the SCN (Rivkees and Lachowicz, 1997).

In the dorsal striatum, dopaminergic input is required for proper modulation of PER2 (Hood et al., 2010), and DA receptors regulate clock gene expression in the striatum *in vitro* (Imbesi et al., 2009). There is evidence that the D1 receptor affects the PER2 protein in the retina (Ruan et al., 2008). Activation of the D2 receptor induces Per1 transcription through recruitment of the CLOCK: BMAL1 heterodimer, an effect that has been shown to be specific to neurons (Yujnovsky et al., 2006).

CRY expression is also required for nocturnal activity in mutants with high DA signaling. Increased DA signaling acts through CRY to drive the nocturnal behavior of Clk mutants. The higher levels of CRY are likely due to post-transcriptional regulation, as the expression of cry mRNA is unaltered (Kumar et al., 2012).

The basal ganglia motor areas, including the striatum, pallidum, subthalamic nucleus (STN) and substantia nigra (SN), are involved in a number of parallel, functionally segregated cortical–subcortical circuits (Groenewegen, 2003). A major role of the SN, the major DA producing center, has been shown to play a regulatory role in both central and peripheral circadian rhythms (Hood et al., 2010; Korshunov et al., 2017). In addition, DA synthesis, release, and signaling are regulated in a circadian manner (Doyle et al., 2002; Chung et al., 2014; Radwan et al., 2018).

The SN, along with the STN, are key brain areas implicated in Parkinson’s disease (PD) and progressive supranuclear palsy, both of which are associated with dysregulation of the dopaminergic system and subsequent disruption of circadian rhythms (Witting et al., 1990; Barrot, 2014).

The characterization of clock genes in these regions in diurnal species, which are evolutionarily closer to humans, may help to functionally correlate circadian signals with motor circuits involved in motor control under both normal and pathological conditions. A deeper understanding of the relationship between motor areas and the presence of these proteins could contribute to the development of new chronotherapeutic strategies for psychiatric and neurodegenerative disorders. Therefore, the aim of this study was to characterize the expression of Per1, Per2 and Cry1 in the SN and STN of a diurnal primate species.

## 2 Materials and methods

### 2.1 Sapajus apella

In the present study, brain slices from six adult male tufted capuchin monkeys (*Sapajus apella*) (2 to 3 kg) of the same age and weight, without visible motor changes, without history of previous diseases, in physiological condition, were obtained from the Tufted Capuchin Monkey Breeding Center of the State University of São Paulo (UNESP), Araçatuba, SP, Brazil. All procedures in this study followed the “Guidelines for the Care and Use of Mammals in Neuroscience and Behavioral Research (2003)” and were approved by the local ethics committees no. 538/2019. The local ethics committees (11/2022, CIAEP-01.0218.2014) approved the use of encephalic slices from capuchin monkeys (*Sapajus apella*). These animals were housed in natural light and fed a controlled diet, with water was provided *ad libitum*. Sunrise at 06:00 AM was considered time 0 (ZT0) and sunset at 18:00 PM (ZT12). Animals were anesthetized with sodium thiopental (30 mg/kg, i.p.) and sacrificed at two different times (ZT10 and ZT19, n = 3 per ZT). Perfusion was performed with a sequence of saline (0.9%) and paraformaldehyde (4%) in 0.1 M sodium acetate buffer and paraformaldehyde (4%) in 0.1 M sodium borate buffer. Brains were cryoprotected, cryosectioned into 30 μm coronal slices, and stored in 10 series in an antifreeze solution at −20°C until immunohistochemistry and Nissl staining were performed.

### 2.2 Immunofluorescence staining

For immunofluorescence staining, encephalic sections were incubated separately with the primary antibodies Per1 (1:500, Santa Cruz, United States), Per2 (1:500, Santa Cruz, USA), and Cry1 (1:500, Santa Cruz, United States). The secondary fluorescent antibody Alexa 488 (1:200, Jackson ImmunoResearch) was then used. To determine cytoarchitecture, sections were stained with Nissl or 4′,6′-diamidino-2-phenylindole (DAPI) stain (Sigma Chemical, cod. D9542 Sigma, St. Louis MO, United States). Negative staining controls were performed by adding Per1 (E-8) blocking peptide (Santa Cruz Biotechnology, sc-398890 P, TX, United States) and Per2 control/blocking peptide #1 Per2 (1-P) (Alpha Diagnostic International, Inc., TX, United States) to the primary incubation solution of Per1 and Per2 antibodies, which blocked Per1 and Per2 staining. For Cry1, the negative antibody control lacked primary antibody. Under these conditions, staining was completely abolished. To ensure that sample differences did not reflect different efficiencies of immunohistochemical labeling, brain sections from the two different Zeitgeber times (ZTs) were processed and incubated in the same solution at the same time. Slides were analyzed under an Olympus BX50 microscope and images were captured using CellSens software (United States). The demarcation of the analyzed areas was made using “A Stereotaxic Atlas of the Brain of *Cebus Monkey* (*Cebus apella*)” (Manocha et al., 1968) and “The Rhesus Monkey Brain in Stereotaxic Coordinates” (Paxinos et al., 1999). Cell quantification was performed using Image J software in three similar coronal sections for all animals as representative of the anteroposterior extent of the SN and STN.

### 2.3 Data analysis

Coronal sections from each animal that were similar across animals (representing the same rostrocaudal level) were then processed for each antibody. Each coronal section (from all six animals) was analyzed under a light field (Olympus BX50 microscope) and images were captured using cellSens software (United States). The images were then analyzed and all visible Per1-IR, Per2-IR, Cry-IR neurons of the SN and STN were counted in each image. Only cells with visible nuclei were counted. The number of cells counted in the coronal sections was representative of the anteroposterior extent (at the rostral level, mid and caudal) of the SN and STN of each animal was used. Data were analyzed using SPSS Statistics 28.0 (SPSS Inc., Chicago, IL, USA). Normal distribution of the data was checked using the Shapiro–Wilk test. Data were expressed as median (interquartile range 25–75%) due to the non-normal distribution found in the vast majority of variables. Comparisons between groups were made using the Kruskal-Wallis test followed by Dunn’s multiple comparison test.

## 3 Results

### 3.1 SN and STN

**Figure 1:**
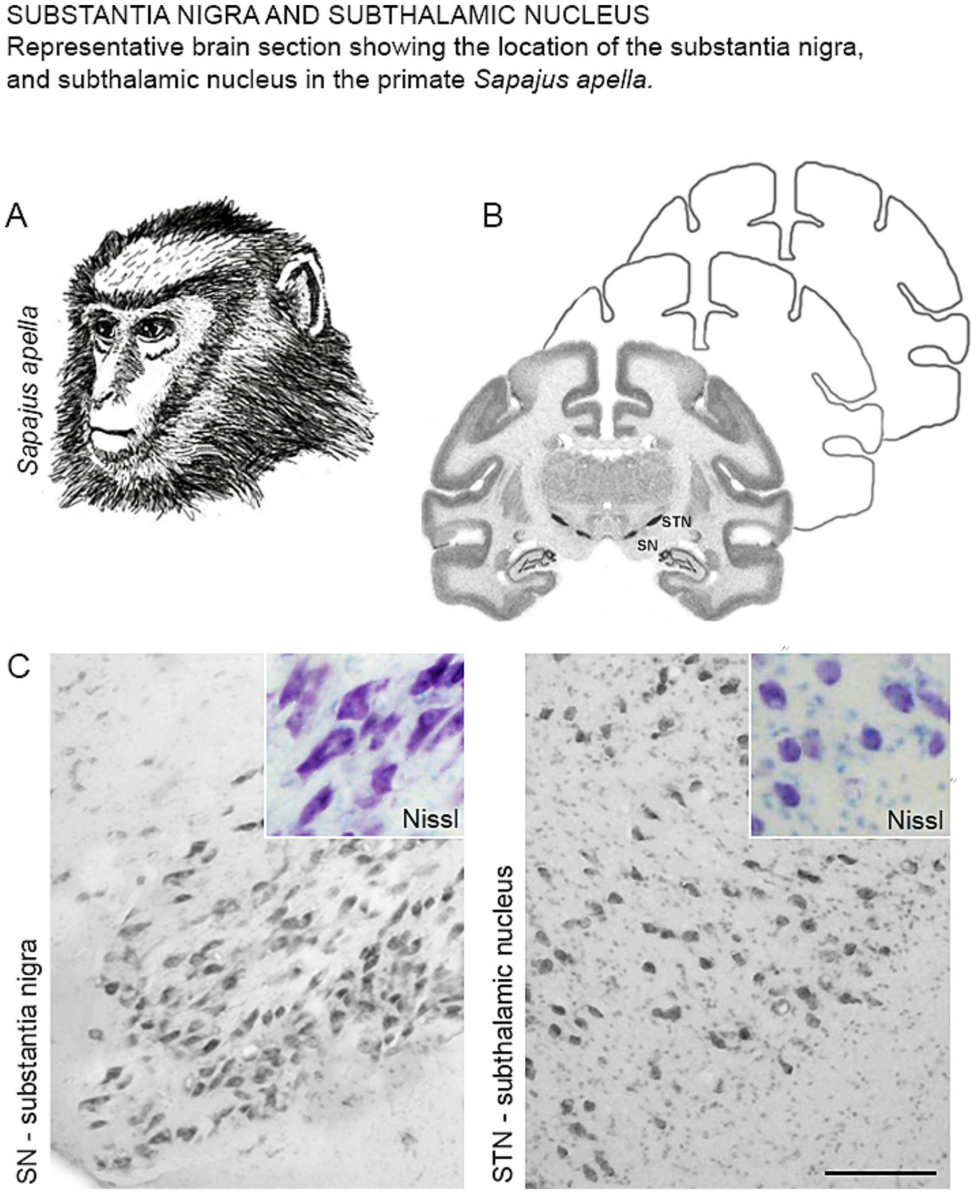
(A) Illustrative drawing of the primate *Sapajus apella*. (B) Representative diagram of a frontal section showing the location of the SN and STN with Nissl staining. (C) Photomicrographs of the coronal section of the SN and STN showing neurons with Nissl staining in *Sapajus apella*.

The characterization of the SN and STN in the primate *Sapajus apella* (Figure 1A), using the Nissl staining technique (Figure 1B), revealed that the SN is located in the posterior (dorsal) mesencephalon at an oblique angle, anterior (ventral) to the midbrain tegmentum. Nissl staining predominantly highlighted the magnocellular neurons within the SN (Figure 1C, SN). The STN was found superior to the SN and the midbrain tegmentum, and caudal to the hypothalamus. Most neurons in this region displayed an ovoid cell body morphology (Figure 1C, STN).

### 3.2 SN

**Figure 2:**
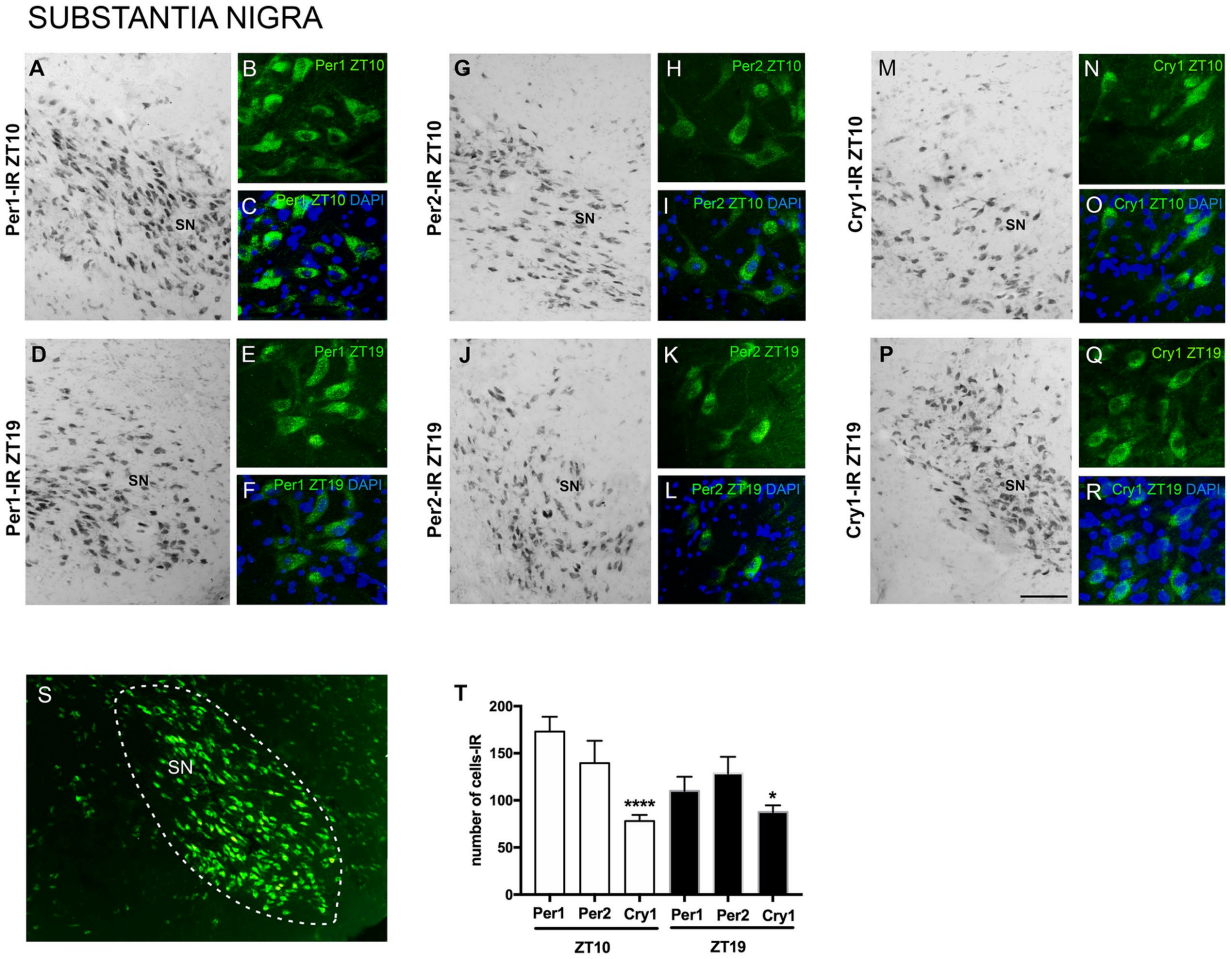
Distribution of Per1, Per2, and Cry1-IR cells in the SN of encephalic slices of the primate *Sapajus apella*. Immunofluorescence photomicrographs of the coronal section of the SN showing Per1-IR the ZT10 (A–C) counterstained with DAPI (blue) (C) and in the ZT19 (D–F) counterstained with DAPI (blue) (F); Per2-IR in the ZT10 (G–I,) counterstained with DAPI (blue) (I), and in the ZT19 (J–L) counterstained with DAPI (blue) (L); Cry1-IR cells in the ZT10 (M–O) counterstained with DAPI (blue) (O), and in the ZT19 (P–R) counterstained with DAPI (blue) (R). In S, delimitation of the SN. In T, the graph shows the median with interquartile range of the number of cells IR for each clock gene, * means that in the ZT10, Cry1 ≠ Per1, *p* < 0.0001 and Cry1 ≠ Per2, *p* < 0.01 (*N* = 3 per ZT). Bar = 100 μm.

The results showed Per1, Per2, and Cry1 labeling in SN neurons at both ZTs. Immunoreactivity (IR) was observed in the nuclear and cytoplasmic regions of SN neurons, with no labeling detected in dendrites or axons. Specific Per1, Per2, and Cry1 labeling was observed at the level of the SN, while adjacent areas showed no labeling, providing clear anatomical delimitation of this region across the different animals analyzed. This indicates the specificity of Per1, Per2, and Cry1-IR within this neuronal population at this level of the anteroposterior axis of the brain (Figure 2).

Considering the number of cells in the representative sections of the anteroposterior axis of the SN, there was a higher number (*p* = 0.0001) of Per1-IR cells [337.0 (292.8–371.0)] and Per2-IR cells [294.0 (240.0–333.5)] when comparing with Cry1-IR [156.5 (129.8-163.3)] in the ZT10 (Figure 2).

### 3.3 STN

**Figure 3:**
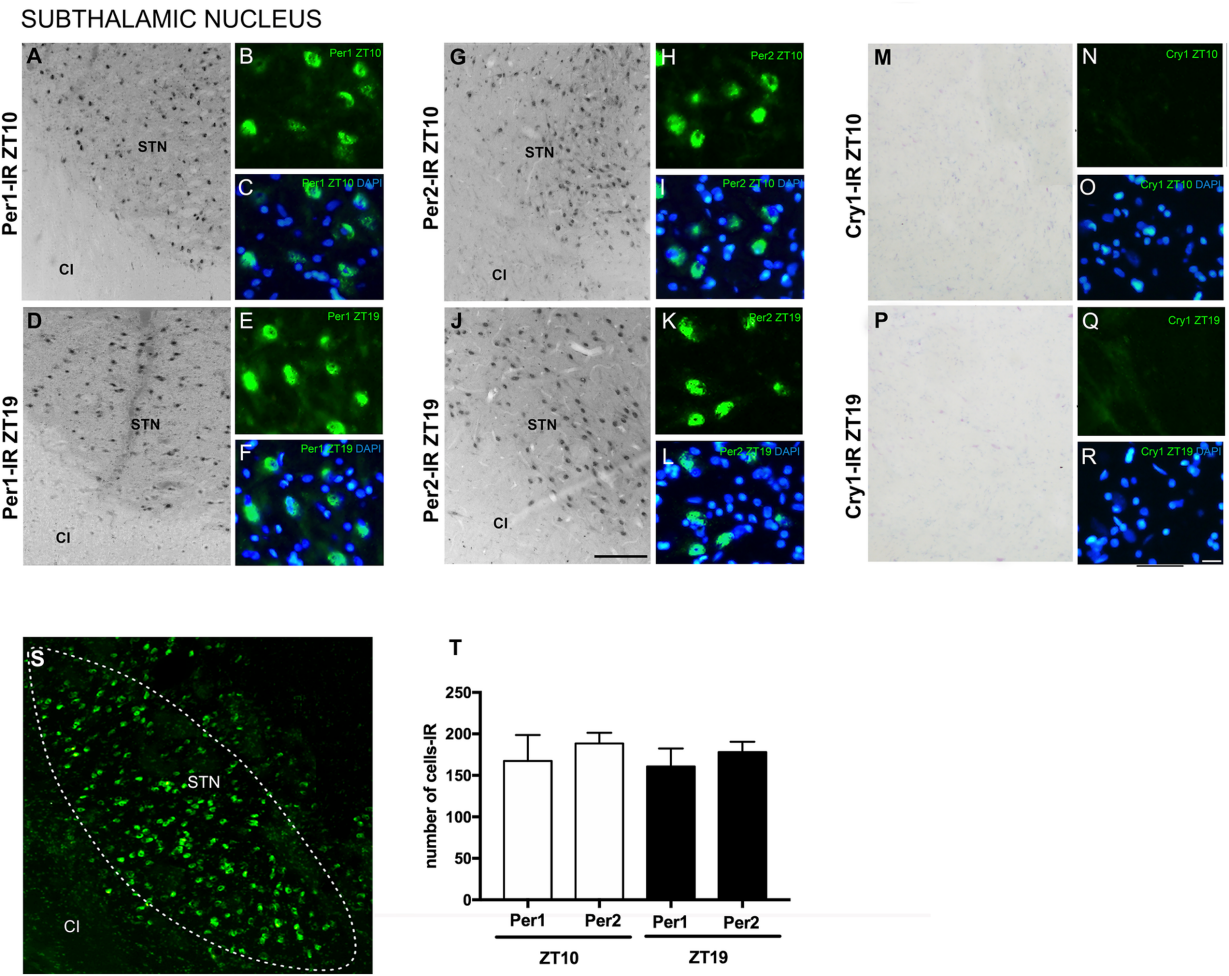
Distribution of Per1, Per2, and Cry1-IR cells in the STN in encephalic slices of the primate *Sapajus apella*. Immunofluorescence photomicrographs of the coronal section of the STN showing Per1-IR in the ZT10 (A–C) counterstained with DAPI (blue) (C) and at ZT19 (D–F) counterstained with DAPI (blue) (F); Per2-IR in the ZT10 (G–I) counterstained with DAPI (blue) (I), and in the ZT19 (J–L) counterstained with DAPI (blue) (L); No Cry1-IR neurons were observed in analyzed (M–R). In (S), delimitation of the STN. In (T), graph showing the median with interquartile range of the number of cells IR for Per1 and Per2 (*N* = 3 per ZT). Capsule internal (CI), and STN. Bar = 100 μm.

The analyses revealed immunoexpression of Per1 and Per2 in the STN at both ZTs. Per1 and Per2 labeling was observed in both oval and polygonal cell bodies. There was no significant difference in the number of cells expressing Per1 compared to those expressing Per2 at either ZT. Cry1-IR labeling was absent in the STN and the areas adjacent to the nucleus at both ZTs, indicating a higher specificity of Per1 and Per2 in this neuronal population (Figure 3).

## 4 Discussion

It is increasingly understood that the presence of circadian clock genes in areas related to motor behavior, such as the basal ganglia, indicates a possible relationship between the circadian system and the dopaminergic system (Hood et al., 2010; Verwey et al., 2016). These areas have implications for many functional and behavioral aspects, ranging from motor control and endocrine release to higher order processing such as cognitive processes (Wise, 2004).

In this study, we investigated whether the clock proteins Per1, Per2 and Cry1 are present in the SN and STN along the anteroposterior extent of these nuclei in the primate *Sapajus apella*.

The observed immunoexpression of Per1, Per2, and Cry1-IR in the nuclear and cytoplasmic regions of SN neurons, with no labeling in dendrites or axons, along with similar findings in the STN where labeling was present in oval and polygonal neuronal cell bodies, is consistent with expectations based on the literature. This intracellular localization supports the development of the negative feedback loop in the transcriptional control of clock genes, indicating the presence of these proteins in both the nucleus and cytoplasm, as reported by Vielhaber et al. (2001) and Hirayama and Sassone-Corsi (2005).

The observed expression of Per1-IR, Per2-IR, and Cry1-IR in the SN of the primate *Sapajus apella* highlights a potential bidirectional relationship between clock genes and neuronal activity of the dopaminergic system in this diurnal species (Guissoni Campos et al., 2015).

Per1 and Per2 were also expressed in the STN at both analyzed ZTs. In contrast, no Cry1-IR staining was observed in the STN at either ZT, which differs from findings in *Drosophila melanogaster*, which is generally diurnal, where Cry1 expression correlates with nocturnal activity (Kumar et al., 2012).

When comparing clock gene expression between mice and baboons, Bmal1 and Per1 showed peaks of expression in the baboon in the evening and morning, respectively, whereas in mice the peaks occurred in the morning and evening, respectively. However, in the case of Cry1 expression in the baboon, the median phase of Cry1 expression was in the late afternoon, whereas in mice its expression is delayed by only 7 h, peaking after midnight (Mure et al., 2018).

Based on these data, the lack of Cry1 expression observed in *Sapajus apella* may indicate potential limitations or artifacts associated with Cry1 in the STN, or that its expression may be dependent on other times of day. These results should be examined at other times of day to confirm this.

The relationship between clock genes and circadian rhythms has been suggested by studies showing circadian patterns in neural activity disturbances within the globus pallidus internus and STN in individuals with PD, with fluctuations occurring during different sleep stages (van Rheede et al., 2022).

In rodents, Per2 exhibits day/night variations in dorsal striatal neurons, with higher expression during the light phase (Hood et al., 2010), and increased expression in the SN during the night (Bussi et al., 2014). The discrepancies between our findings and those in other species may be due to the specific ZTs analyzed or interspecific differences, highlighting the importance of conducting studies in diurnal species, especially primates.

Circadian rhythmicity in rodent motor areas is thought to result from the influence of DA on clock protein expression, which may also affect DA synthesis, release, and signaling (Doyle et al., 2002; Chung et al., 2014; Korshunov et al., 2017). These findings contribute to the understanding of regulation of the sleep–wake cycle by DA action in the hypothalamic and mesolimbic pathways (Oishi and Lazarus, 2017).

The characterization of clock genes and the correlation between daily fluctuations in DA function and circadian activity have been demonstrated in various areas, including the retina, olfactory bulb, striatum, midbrain, and hypothalamus (Yujnovsky et al., 2006; Hood et al., 2010; Chung et al., 2014; Mendoza and Challet, 2014), suggesting a bidirectional relationship between circadian rhythms and motor areas (Kafka et al., 1986; Golombek et al., 2014).

These associations are also implicated in pathological conditions, particularly neurodegenerative diseases in which sleep disturbances and circadian dysfunction are prominent, such as PD, Huntington’s disease, and Alzheimer’s disease (Mattis and Sehgal, 2016; Herzog-Krzywoszanska and Krzywoszanski, 2019; Leng et al., 2019).

Circadian disruption in PD has been associated with a wide range of symptoms, including sleep–wake disturbances, autonomic dysregulation, temperature imbalance, and motor fluctuations (Zuzuárregui and During, 2020). Under normal conditions, changes in STN neuronal activity can be observed during the day and night. STN neurons switch from a random discharge pattern during wakefulness to a burst pattern during slow-wave sleep (SWS) without changing their average firing rate. This discharge pattern appears to depend on coincident cortical activity (Urbain et al., 2000).

The presence of Per1, Per2 and Cry1 in the SN, together with Per1 and Per2 in the STN, may suggest a circadian influence on local motor functions. The expression of clock genes in the caudate and putamen nuclei of rats has already been correlated with locomotor activity (Masubuchi et al., 2000), which may be relevant to locomotor and reward mechanisms in a diurnal species (Yujnovsky et al., 2006; Hampp et al., 2008).

## 5 Conclusion

The presence of clock proteins in the SN and STN of diurnal primates suggests a relationship between clock gene proteins, the dopaminergic system, and areas associated with motor behavior.

## Data Availability

Data and any supplementary material related to this article can be obtained from the corresponding author upon request.
